# The Effectiveness of a Mindfulness-Based Intervention Integrated with Physical Therapy (MIND-PT) for Postsurgical Rehabilitation After Lumbar Surgery: A Protocol for a Randomized Controlled Trial as Part of the Back Pain Consortium (BACPAC) Research Program

**DOI:** 10.1093/pm/pnac138

**Published:** 2022-09-07

**Authors:** Julie M Fritz, Daniel I Rhon, Eric L Garland, Adam W Hanley, Tina Greenlee, Nora Fino, Brook Martin, Krista B Highland, Tom Greene

**Affiliations:** Department of Physical Therapy & Athletic Training, The University of Utah, Salt Lake City, Utah; Department of Rehabilitation Medicine, Brooke Army Medical Center, San Antonio, Texas; Department of Rehabilitation Medicine, Uniformed Services University of Health Sciences, Bethesda, Maryland; College of Social Work, The University of Utah, Salt Lake City, Utah; College of Social Work, The University of Utah, Salt Lake City, Utah; Department of Rehabilitation Medicine, Brooke Army Medical Center, San Antonio, Texas; Department of Population Health Sciences, The University of Utah, Salt Lake City, Utah; Department of Orthopedics, School of Medicine, The University of Utah, Salt Lake City, Utah; Department of Orthopedics, School of Medicine, The University of Utah, Salt Lake City, Utah; Defense and Veterans Center for Integrative Pain Management, Department of Anesthesiology, Uniformed Services University, Bethesda, Maryland; Henry M. Jackson Foundation for the Advancement of Military Medicine, Rockville, Maryland, USA; Department of Population Health Sciences, The University of Utah, Salt Lake City, Utah

**Keywords:** Lumbar Spine, Surgery, Clinical Trial, Physical Therapy, Mindfulness

## Abstract

**Background:**

Improving pain management for persons with chronic low back pain (LBP) undergoing surgery is an important consideration in improving patient-centered outcomes and reducing the risk of persistent opioid use after surgery. Nonpharmacological treatments, including physical therapy and mindfulness, are beneficial for nonsurgical LBP through complementary biopsychosocial mechanisms, but their integration and application for persons undergoing surgery for LBP have not been examined. This study (MIND-PT) is a multisite randomized trial that compares an enriched pain management (EPM) pathway that integrates physical therapy and mindfulness vs usual-care pain management (UC) for persons undergoing surgery for LBP.

**Design:**

Participants from military treatment facilities will be enrolled before surgery and individually randomized to the EPM or UC pain management pathways. Participants assigned to EPM will receive presurgical biopsychosocial education and mindfulness instruction. After surgery, the EPM group will receive 10 sessions of physical therapy with integrated mindfulness techniques. Participants assigned to the UC group will receive usual pain management care after surgery. The primary outcome will be the pain impact, assessed with the Pain, Enjoyment, and General Activity (PEG) scale. Time to opioid discontinuation is the main secondary outcome.

**Summary:**

This trial is part of the National Institutes of Health Helping to End Addiction Long-term (HEAL) initiative, which is focused on providing scientific solutions to the opioid crisis. The MIND-PT study will examine an innovative program combining nonpharmacological treatments designed to improve outcomes and reduce opioid overreliance in persons undergoing lumbar surgery.

## Background and Rationale

Chronic pain is a common and consequential condition for civilian and military populations. Low back pain (LBP) is the most common chronic pain diagnosis and the most common diagnosis associated with an opioid prescription in both military and civilian health care [1–3]. Because of its high prevalence and its contribution to concerns about opioid overprescribing, improving the management of chronic LBP is a priority across health care settings [4–6].

A small percentage of persons with chronic LBP receive surgery, but many have used opioids long term for pain management [7–9]. Recent studies indicate that a third to half of persons undergoing elective spine surgery have used opioids long term, and most will receive at least one postoperative prescription [8, 10]. Despite optimism from providers and patients that surgery will eliminate the need for long-term opioid use, most persons who used opioids long term before surgery persist in use, even 2 years after surgery, some at increased dose [10–12]. Presurgical opioid use is also associated with worse functional outcomes, increased rates of complications, and higher medical costs [11, 13–15]. Persistent postsurgical opioid use is associated with increased risk of interventional pain procedures, re-operation, opioid-related adverse events, and higher medical costs [11, 16]. These findings indicate that outcomes after lumbar surgery will not improve without a comprehensive strategy for better pain management that prepares patients to manage pain without overreliance on opioids.

Efforts to reduce opioid overreliance after spine surgery have focused primarily on limiting opioid prescribing via regulatory and payer policies [17, 18]. The Army Surgeon General’s Pain Management Task Force [19], the U.S. Centers for Disease Control and Prevention [20], and the Federal Interagency National Pain Strategy [21] all emphasize the critical role of nonpharmacological care to improve pain management and reduce opioid overreliance. Nonpharmacological pain management for persons undergoing thoracolumbar, lumbar, or lumbosacral (herein “lumbar”) surgery has not been sufficiently studied as a strategy to both improve patient-centered outcomes and reduce risk for prolonged postsurgical opioid use [22]. Furthermore, there has been inadequate attention to the critical role of psychological factors in the experience of chronic pain in a comprehensive approach to pain management pathways for persons undergoing lumbar surgery.

**Figure 1.**
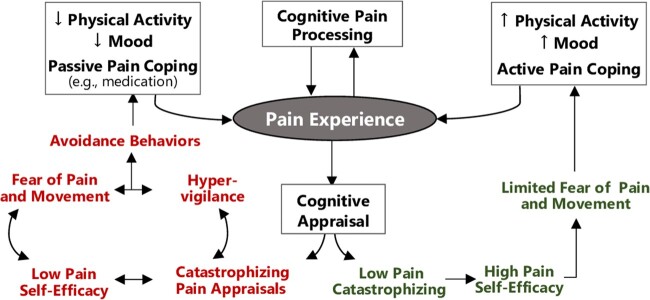
Biopsychosocial paradigm relating cognitive and emotional responses to pain to outcomes.

The present study is grounded in a biopsychosocial model of chronic pain and factors contributing to poor clinical outcomes and persistent opioid use (Figure 1). The model recognizes that pain’s aversive nature elicits powerful cognitive and emotional responses that feed back to modulate pain perceptions and coping behaviors [23–25]. Patients making catastrophic appraisals, or assumptions of the worst imaginable outcomes, about pain typically have less confidence in their ability to self-manage pain (i.e., low self-efficacy) [26], leading to fear and heightened attentional bias to perceived pain-related threats (i.e., hypervigilance), all of which promote activity avoidance [27]. Persistent pain-related catastrophizing can reduce the ability to engage in adaptive cognitive regulation of pain and primes the person for an exacerbated pain experience [28], which can reinforce catastrophic appraisals in a self-perpetuating cycle. Although opioids have limited analgesic effect [29, 30], their use can persist as a way to manage pain-related emotions for persons with chronic pain. Thus, actual or anticipated pain can trigger an automatic reaction (taking opioids), and unconscious habit replaces conscious choice as the primary determinant of opioid use [31]. Patients approaching surgery might have been caught in this cycle for years.

The relevance of the biopsychosocial model to patients receiving spine surgery is supported by evidence that pain catastrophizing, low self-efficacy, and hypervigilance predict poor postsurgical outcomes [32–34]. These factors also predict long-term postsurgical opioid use [35, 36]. Spine surgery can exacerbate catastrophic thinking [37, 38], especially if patients have unrealistic recovery expectations that go unmet [39, 40]. Nonpharmacological treatments have the potential to disrupt the cycle of catastrophic appraisals, low self-efficacy, and hypervigilance that can reinforce overreliance on opioid medication. Positive effects of physical therapy (PT) for LBP are mediated through changes in pain catastrophizing and self-efficacy [41–44]. Mindfulness, which involves the generation of temporary states of monitoring mental experiences and sensory information without evaluation, reduces pain attentional bias and disentangles the pain experience from associated emotions and catastrophic appraisals [45]. The effects of mindfulness on reducing chronic pain severity are mediated by shifting from emotional to sensory processing of the pain experience [46, 47], and mindfulness-based interventions can increase self-awareness and self-regulation of conditioned, automatic habits like opioid use [48, 49] and reduce reactivity to opioid-related cues (e.g., the sight of an opioid pill bottle) [50]. Given these complementary mechanisms, integrating PT and mindfulness techniques might be particularly beneficial for persons with chronic pain undergoing surgery, but this premise has not been evaluated.

The purpose of the present article is to describe the MIND-PT study, which will evaluate a nonpharmacological strategy that integrates PT and mindfulness in an enriched pain management pathway for patients undergoing lumbar surgery. The enriched pathway threads consistent messages and techniques focused on developing non-opioid pain coping strategies from the presurgery to postsurgery periods to improve surgical outcomes and reduce risk of persistent opioid use. We will compare the enriched pathway to usual-care pain management.

## Methods

### Study Rationale and Objective

Evidence supports the effectiveness of PT and mindfulness for patients with nonsurgical chronic LBP [51]. These treatments have not been integrated and evaluated as a comprehensive pain management pathway for persons undergoing lumbar surgery. Integrating PT and mindfulness might be particularly effective at disrupting the self-reinforcing cycle of pain, catastrophic appraisal, pain-related emotional reactions, hypervigilance, and unconscious behavioral response that can contribute to persistent opioid use. Finally, developing nonpharmacological pain coping strategies for patients undergoing spine surgery is often not explicitly and consistently addressed during the surgical experience, nor are such strategies addressed in postsurgical pain management consensus recommendations [52, 53]. Our project examines an innovative strategy to integrate mindfulness and PT into an enriched pain management (EPM) pathway for patients undergoing lumbar surgery. The long-term goal of this trial is to develop a scalable physical therapist–driven EPM intervention. Physical therapists are typically involved in presurgical and postsurgical care for patients undergoing lumbar surgery, but their efforts often lack standardization, fail to incorporate mindfulness techniques, and do not explicitly focus on developing nonpharmacological pain coping strategies to reduce opioid overreliance.

### Study Design

**Figure 2.**
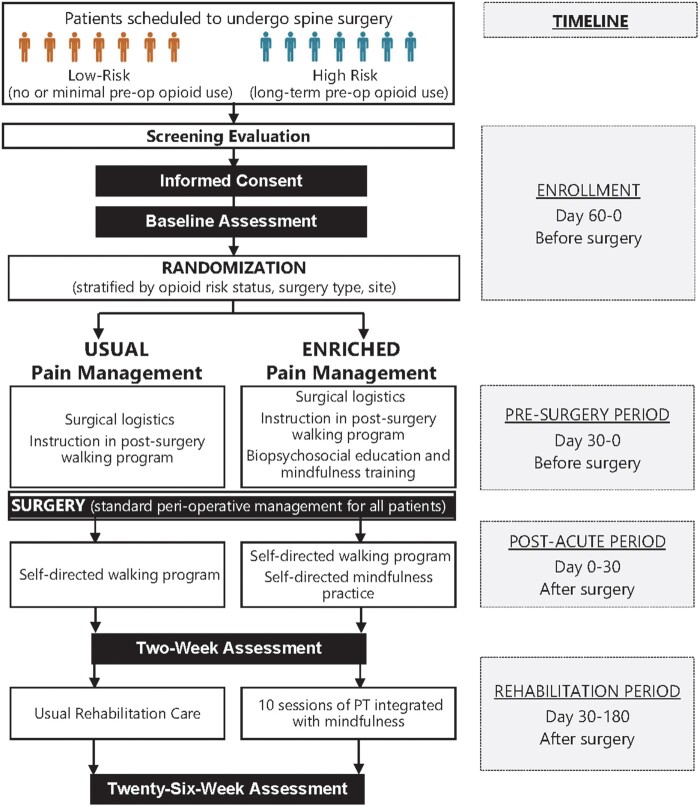
Study diagram and timeline.

The MIND-PT study is a two-arm, parallel group, individual-randomized trial (Figure 2). The trial is registered with clinicaltrials.gov (unique identifier NCT04770480). We will recruit persons scheduled for lumbar surgery in a participating facility. Individuals who provide consent will be randomized before surgery either to the EPM pathway that integrates PT and mindfulness or to usual-care pain management (UC). Presurgical and postsurgical care is based on the randomized group assignment with consideration of surgery type (fusion or nonfusion). The surgical procedure itself and perioperative care, including the use of prescription opioids, do not differ by treatment group and are based on the standard procedures at the participating facility and discretion of the surgeon. Assessments occur at enrollment (before surgery) and 2 and 26 weeks after surgery. Persons who do not undergo surgery for any reason will be removed from the study. Our hypotheses are that the EPM pathway will be superior to UC in the overall cohort for the primary outcome of pain impact. We further hypothesize that the EPM pathway will be superior to UC for the outcomes of pain impact and opioid discontinuation in predefined subgroups who could be at elevated risk of postsurgical long-term opioid use, indicating a need to explore treatment effect heterogeneity across different risk levels.

### Study Population

The MIND-PT study is designed to recruit a representative cohort of active-duty military, family members of active-duty personnel, and retirees who are TRICARE beneficiaries scheduled for lumbar surgery in a military or civilian hospital. Specific eligibility criteria are outlined in Table 1. Participants will also be identified as meeting criteria for long-term opioid use before surgery, as these patients could be at the highest risk of postsurgical long-term opioid use [10, 12].

**Table 1.** Eligibility criteria for the MIND-PT trial

Inclusion Criteria	Exclusion Criteria
1. Active-duty military or member of Reserves or National Guard on active duty, family member of active-duty personnel, or other TRICARE beneficiary receiving care in a participating facility.	1. Indication for surgery is infection, fracture, tumor, trauma, or other indication requiring emergency surgery.
2. Age 18–75 years.	2. Surgical procedure is a revision, or individual has undergone a lumbar surgical procedure in the past year.
3. Scheduled to undergo lumbar spine surgery within the next 60 days. Surgery may be a microdiscectomy, discectomy, or laminectomy with or without fusion, including lateral, transforaminal, posterior, or anterior approach for 1–4 lumbar levels. Procedure may be performed in a military or civilian hospital.	3. Contraindication to participation in postoperative exercise program, including severe orthopedic injury limiting mobility, wheelchair dependency, neurological disorder impacting mobility, reliance on supplemental oxygen for daily activity, etc.
4. Anticipates ability to attend treatment sessions over a 16-week period after the surgical procedure, with no planned absence of 2 weeks or more for training, vacation, or any purpose.	4. Pending a medical evaluation board, discharge from the military for medical reasons, or pending or undergoing any litigation for an injury.
5. Able to speak and read English well enough to provide informed consent, follow study instructions, and independently answer surveys	

### Screening, Recruitment, and Randomization

Recruitment will occur among patients scheduled for lumbar surgery, including fusion, laminectomy, decompression, or discectomy, at recruitment sites. Potential participants will be identified as they schedule for surgery. When a patient is scheduled for surgery, they are entered into a pathway that involves follow-up assessments and a final presurgical visit. The study team will maintain communication with the surgical team to receive referrals of patients to the study. In addition to efforts for direct referral, we will secondarily identify potential participants from the surgical roster for future surgeries from the surgical scheduling system. A Health Insurance Portability and Accountability Act (HIPAA) waiver allows approved research staff to review the appointment list in the Military Health System electronic health record. Scheduled patients may be notified of the study and their potential eligibility. Interested individuals will meet with a site research assistant who will facilitate the informed consent process.

Treatment assignment will occur after consent and baseline assessment are complete. Individual participant randomization with 1:1 distribution will be administered with the REDCap randomization module (Vanderbilt University, Nashville, TN, USA). A study statistician created a randomization allocation table by using blocked randomization with block sizes of 4 or 6. Randomization is stratified by recruitment site, presurgery opioid use (yes/no), and type of surgery (planned with or without fusion). The allocation table was prepared with SAS software version 9.4 (SAS Institute Inc., Cary, NC, USA).

### Participating Sites

Recruitment sites are orthopedic and neurosurgery clinics at Brooke Army Medical Center and Wilford Hall Ambulatory Surgical Center at the San Antonio Military Medical Health System in San Antonio, Texas; Madigan Army Medical Center in Tacoma, Washington; and Tripler Army Medical Center in Honolulu, Hawaii.

### Interventions

Study interventions are provided across the presurgical and postsurgical periods in three phases: the presurgery period, the post-acute period, and the rehabilitation period, with time frames outlined in Figure 2.

#### Usual-Care Pain Management

Participants randomized to the UC group will receive care consistent with current practice at participating military treatment facilities. Participants in the UC group will receive a single presurgical session. This session will provide site-specific logistic information and standard written information on proper body mechanics; postsurgical precautions for lifting, twisting, and bending; sleeping positions; and a walking program to begin in the post-acute period. Specifically, participants will be advised to begin progressive mobility with a goal of walking for about 10 minutes, progressing to 20–30 minutes two to four times per day by the time of the postoperative follow-up with the surgeon.

The rehabilitation period will begin after the participant’s postsurgical follow-up with the surgeon and completion of the 2-week follow-up assessment. The rehabilitation period is expected to begin later for patients undergoing surgery with fusion. PT or other services provided in the rehabilitation period will be based on site-specific procedures and surgeon preferences.

#### Enriched Pain Management

Participants randomized to the EPM group will receive treatments across the presurgery, post-acute, and rehabilitation periods that are grounded in the biopsychosocial model informing the project (Figure 1). Participants will receive a single presurgical session. This session will provide site-specific logistical information, written instruction in body mechanics and precautions, and a postsurgery walking program, as in the UC group. Participants in the EPM group also receive a 30-minute mindfulness component during the presurgical session to provide education in a biopsychosocial model of chronic pain, an introduction to the role of mindfulness in recovery from surgery, and instruction in a brief mindfulness practice for use during the perioperative and post-acute periods. Participants will be advised to begin progressive mobility as described for the UC group.

The rehabilitation period will begin after the participant’s postsurgical follow-up visit and completion of the 2-week follow-up assessment. The rehabilitation period is expected to begin later for patients undergoing surgery with fusion, with the timing based on postsurgical restrictions and surgeon preferences. The rehabilitation period may continue for up to 180 days after surgery to allow sufficient time to complete the intervention even if the start is delayed.

Participants in the EPM group will receive 10 weekly sessions of PT in the rehabilitation period. Standard PT exercises to enhance strength, flexibility, and endurance will be provided, along with integrated further instruction, psychoeducation, and practice of mindfulness techniques for nonpharmacological pain coping. Mindfulness techniques are based on the Mindfulness-Oriented Recovery Enhancement (MORE) program [54, 55]. The outline of the 10-session protocol for the EPM group in the rehabilitation period is provided in Table 2. Integration of mindfulness begins with a review of the mindfulness instruction provided before surgery. Patients are reminded that the goal of mindfulness is to help them cope with pain and stress during the recovery process by focusing the mind on the present experience. Instruction in body scan meditation is intended to reinforce the concept of mindfulness. In session 4, participants are taught about the concept of automaticity in chronic pain (e.g., maladaptive and habitual pain coping behaviors like inactivity or taking more medication than intended, etc.) that can be triggered subconsciously by negative emotions. Session 6 introduces the concept of mindful reappraisal as means of coping with negative emotions, such as fear of pain and catastrophizing. Finally, participants learn about mindful savoring in session 8. Mindful savoring is attentional reorienting as a means of coping with negative emotions and pain through attentional focus on pleasant experiences as a means of amplifying the hedonic value of natural rewards [56]. Patients are provided links to videos of body scan, mindful breathing, and savoring exercises for practice between sessions.

**Table 2.** Outline of 10 PT sessions for participants in the EPM group during the rehabilitation period

SESSION	
1	**PT Assessment**
	**PT Treatment** Review body mechanics and precautions Review and advance walking/mobility program Practice the same mindfulness practice that was introduced at the preoperative visit (should be review) Begin isometric stabilization exercises
2	**Mindfulness Component** Discuss the Nature of Pain (7 minutes) Discuss the Practice of Mindfulness (8 minutes) Instruction in Body Scan Meditation (10 minutes)
	**PT Treatment** Advance walking/mobility, stabilization exercise program Begin stretching and spinal mobility
3	**PT Treatment** Advance walking/aerobic exercise program (mindful walking on the treadmill with pre-recorded mindfulness session) Advance stabilization and stretching / spinal mobility exercise program
4	**Mindfulness Component** Debrief daily mindfulness / body scan practice (10 minutes) Discuss automaticity in coping with pain and stress (15 minutes)
	**PT Treatment** Advance aerobic, stabilization, stretching/mobility exercise programs Begin resistance exercise training
5	**PT Treatment** Mindful walking on the treadmill with pre-recorded mindfulness session Advance aerobic, stabilization, stretching/mobility and resistance exercise programs
6	**Mindfulness Component** Debrief daily mindfulness / body scan practice (7 minutes) Discuss the power of positive reappraisal (10 minutes) Reappraisal example (7 minutes)
	**PT Treatment** Continue/advance aerobic, stabilization, stretching, and resistance exercise program
7	**PT Treatment** Mindful walking on the treadmill with pre-recorded mindfulness session Continue/advance aerobic, stabilization, stretching, and resistance exercise program
8	**Mindfulness Component** Debrief daily mindfulness / body scan practice (10 minutes) Discuss mindful savoring (7 minutes) Mindful savoring exercise practice (10 minutes)
	**PT Treatment** Continue/advance aerobic, stabilization, stretching, and resistance exercise program
9	**PT Treatment** Mindful walking on the treadmill with pre-recorded mindfulness session Continue/advance aerobic, stabilization, stretching, and resistance exercise program
10	**Discharge Planning** Exercise program instruction for continuation after discharge Review mindfulness practices

### Blinding

Participants and clinicians cannot be blinded to study treatments. Randomization assignment will be revealed after the baseline examination is complete to reduce potential bias by either the participant or researchers. Follow-up assessments will be performed by a blinded research assistant or conducted remotely via web-link to self-report outcome measures. Instances of unblinding during an assessment will be recorded on an electronic case report form.

The randomization allocation table was developed before enrollment by statisticians not involved with managing the study databases. Study investigators will remain blinded to participants’ treatment group assignment. Blinding will be broken only in emergency situations for safety reasons, such as a serious adverse event. If necessary, the study statistician will break the blinding. If blinding is broken, the reason will be fully documented and entered on an electronic case report form.

### Treatment Adherence and Fidelity

Participants’ treatment adherence in the EPM group will be evaluated on the basis of attendance at scheduled treatment sessions in the presurgical and rehabilitation periods. For participants in the UC group, adherence will be evaluated on the basis of attendance at the single presurgical session. Additional rehabilitation visits will be recorded from the electronic health record. Provider fidelity to EPM treatment core components will be self-reported on checklists for each session. Checklists will also record off-protocol interventions. For purposes of per-protocol analyses, we will define adherence as occurring when a participant in the EPM group attends the presurgical session and five or more PT sessions in the rehabilitation period. Neither patients nor providers will be removed for nonadherence or poor fidelity.

### Provider Training

Investigators will provide training for the physical therapists providing the study interventions. Physical therapists must be licensed and credentialed to provide care in participating Military Health System sites. An intervention manual has been developed to facilitate training. Providers receive one-day training on the biopsychosocial model of pain; strategies for delivering the mindfulness, reappraisal, and savoring techniques from the MORE intervention; and detailed instruction on the intervention procedures across study periods.

### Ethics and Data and Safety Monitoring

The study protocol has been approved by the Regional Health Command–Central and University of Utah institutional review boards. An independent data and safety monitoring board will meet at least annually to monitor the course of the study and provide safety oversight. Adverse events related to participation in the study might be identified during intervention or follow-up sessions and will be recorded and reported in compliance with the procedures established by the institutional review boards and data and safety monitoring board.

### Schedule of Assessments

Assessments are conducted at baseline (before randomization and before surgery) and at weeks 2 and 26 after enrollment (Table 3).

**Table 3.** Schedule of assessments for the MIND-PT trial

Outcome	Screening	Baseline Assessment	Postsurgery Assessment at 2 Weeks After Surgery	Postsurgery Assessment at 26 Weeks After Surgery
		(days ^−^30–0)	(days 10–30)	(days 160–200)
Informed consent	X			
Eligibility criteria	X			
Demographics		X		
Medical history		X		
PEG (primary outcome)*		X	X	X
TAPS Substance Use Screener*		X		
PROMIS—Pain Interference short form		X	X	X
PROMIS—Physical Function short form*		X	X	X
PROMIS—Sleep Disturbance short form*		X	X	X
PHQ-2 depression screener*		X	X	X
GAD-2 anxiety screener*		X	X	X
High-impact chronic pain		X		X
Pain self-efficacy		X	X	X
Pain catastrophizing*		X	X	X
Patient global impression of change*				X
Health care utilization for back pain^†^				X
Opioid discontinuation^†^				X

* Indicates an NIH HEAL initiative core domain for assessment of adult chronic pain [56].

† Indicates some aspects of the data element are collected from the electronic health record.

### Outcomes

Outcome measures for the MIND-PT study are compliant with the common data elements established by the National Institutes of Health Helping to End Addiction Long-term (NIH HEAL) initiative. The NIH HEAL initiative identified nine core domains for pain assessment [56] with corresponding questionnaires [57] that are included in this study (Table 3).

#### Primary Outcome

The primary outcome is the Pain, Enjoyment, and General Activity (PEG) Scale at 26 weeks. The PEG includes three items evaluating 1) pain severity on average in the prior week, 2) interference of pain with enjoyment, and 3) interference of pain with general activity. Response options for each item range from 0 to 10, and the PEG score is expressed as the mean of all item scores [58]. The minimally important difference for the PEG is estimated as 1.0 [59]. Variability, assessed as standard deviations, ranges from 2.0 to 2.2 for baseline scores and from 2.5 to 2.8 for change scores in groups receiving an effective intervention [58, 60].

#### Main Secondary Outcome

Time to opioid discontinuation will be the main secondary outcome. Opioid use will be determined from the Pharmacy Data Transaction Service data table of the Military Health System Data Repository. The Military Health System Data Repository contains TRICARE claims for inpatient and outpatient encounters provided in military or civilian facilities. The unique number of prescriptions and total days’ supply will be extracted for all prescriptions involving opioid partial agonists or agonists in categories II, III, or IV. Time to discontinuation will be defined as the days’ duration between the date of surgery and a period of 31 days after surgery with no opioid fill and no additional fills beyond the 31st day. When this definition is met, the 31st day from the last fill is the date of discontinuation [5].

#### Additional Secondary Outcomes

Additional NIH HEAL core outcome domains include Patient-Reported Outcomes Measurement Information System (PROMIS) short forms assessing pain interference (4a), physical function (6b), and sleep disturbance (6a). All PROMIS scores are reported on a T-score metric, with a score of 50 points aligning with the general population mean and a standard deviation of 10 [61]. The General Anxiety Disorder two-item screener (GAD-2) and the Patient Health Questionnaire two-item mood screener (PHQ-2) are used to identify general anxiety and depression, respectively. The GAD-2 and PHQ-2 each have two items with response options ranging from 0 to 3. A score of 3 or more has been used to identify possible cases of generalized anxiety disorder on the GAD-2 [62]. For the PHQ-2, a score of 3 or more is associated with a major depressive episode [63]. The Tobacco, Alcohol, and Prescription Medications and other Substance (TAPS) Tool is a four-item screening for tobacco use, alcohol use, prescription medication misuse, and illicit substance use in the prior year. Each item has five response options ranging from “daily or almost daily” to “never.” Responses other than “never” are considered a positive screen [64]. The Pain Catastrophizing Scale (PCS-6) and Self-Efficacy (PSEQ-4) short-form questionnaires are used to evaluate a participant’s negative cognitive–affective response to anticipated or actual pain and their confidence in performing activities despite pain, respectively [65]. High-impact chronic pain is identified from two questions: 1) “In the past 3 months, how often did you have pain?” and 2) “Over the past 3 months, how often did pain limit your life or work activities?” Responses of “most days” or “every day” to both questions identifies high-impact chronic pain [66]. The single-item Patient Global Impression of Change measure assesses participants’ global impression of change after treatment on the basis of the question, “Since the time period right before I had surgery, my overall pain is …,” with Likert scale response options ranging from (1) “very much worse” to (7) “very much improved.”

### Statistical Methods

Analyses for the project aims are outlined below. Intention-to-treat principles will guide the analyses, with patients evaluated on the basis of randomized assignment regardless of compliance.

#### Aim 1 (Primary)

The primary aim compares the effectiveness of two pain management pathways (UC vs EPM). The primary effectiveness end point will be change in PEG score at 26 weeks after surgery. The primary analysis will be performed by fitting a longitudinal linear model to the PEG scores at baseline and at the 2- and 26-week follow-ups [67]. Application of a longitudinal analysis, rather than performance of separate analyses for each time point, will better account for missing data [68]. The longitudinal model assumes equal mean baseline PEG scores across treatment arms [69]. An unstructured covariance matrix will be used to account for correlation of serial measures in the same subject to avoid imposing specific assumptions about distributions of random effects [70]. The longitudinal model will include fixed effects for randomized treatment group, visit time (as a category variable), and the group×time interaction. The model will also include covariates for the randomization stratification factors and their interactions with visit time. The primary assessment of effectiveness will be based on a linear contrast comparing adjusted mean PEG scores at 26 weeks between groups. Additional linear contrasts will assess effectiveness on the 2-week PEG score and will evaluate whether the magnitude of the effect of EPM compared with UC changes between the 2- and 26-week assessments.

#### Aim 1 (Main Secondary)

Time to opioid discontinuation is the main secondary outcome and is defined as the time between the date of surgery and a period of 31 days after surgery with no opioid fill and no additional fills beyond the 31st day. When this definition is met, the 31st day from the last fill is the discontinuation date [5]. Kaplan-Meier curves with 95% pointwise confidence limits will be constructed to summarize the time to opioid discontinuation in each treatment group. Between-group comparison will use a stratified log-rank test, stratified by the three randomization stratification factors. We will also carry out a companion stratified Cox proportional-hazards regression analysis to express the treatment effect on time to opioid discontinuation as a hazard ratio. The log-rank test and Cox regression analyses will be right-censored at the date of last opioid use assessment for patients with premature loss-to-follow-up and at 26 weeks for patients who complete their 26-week follow-up period without opioid discontinuation.

#### Aim 1 (Other Secondary Analyses)

Similar longitudinal models as described for the primary outcome will be applied for analyses of additional patient-reported outcomes (Table 3). Generalized estimating equations under generalized linear models with negative binomial outcomes models and logarithmic link functions will be used to compare count outcomes, including frequency of opioid use at the 26-week assessment.

#### Aim 1a (Subgroup Analysis)

Subgroup analysis will be performed by repeating the Aim 1 longitudinal analysis within prespecified subgroups based on presurgical opioid risk (high/low) and extending the model by adding a main effect term for opioid risk, as well as the interaction between treatment group and opioid risk. Results of the subgroup analysis will be assessed primarily on the basis of the treatment×subgroup interaction, using a two-sided α = 0.05. We will also add similar main effect and interaction terms to the Cox regression analysis of time to discontinuation of opioid use and to the logistic regression analysis of opioid use at 26 weeks to evaluate treatment effect heterogeneity on the opioid use outcomes.

#### Aim 2 (Mediation Analyses)

We will conduct exploratory mediation analyses to estimate indirect effects that express the extent to which early changes (from baseline to week 2) in self-efficacy and pain catastrophizing mediate the effects of the interventions on PEG scores and opioid discontinuation at 26 weeks [71]. For each potential mediator and each outcome, mediation analysis will be built from two regression models: 1) a regression relating early change in the potential mediator to the randomized treatment group and 2) a regression relating the outcome jointly to the mediators and the randomized treatment groups. For the PEG, the second regression will be carried out with the mixed-effects models previously described for the primary analysis. For opioid discontinuation, the second regression will be carried out with generalized estimating equations for a binary outcome. The second regression will include interaction terms to account for possible moderation of the effects of the randomized treatments by the mediating variable [71]. Both regressions will include baseline covariates to control for confounding factors that could jointly influence the mediator and outcome. Mediation analyses are particularly subject to risk of bias from uncontrolled confounding and require other difficult-to-verify assumptions. Hence, these analyses will be interpreted as exploratory.

#### Procedures for Handling Missing Data

Multiple imputation will be used to assure statistical inferences remain valid in the presence of missing data if missingness follows a missing-at-random mechanism [72]. To protect against bias resulting from association of the probability of missingness on other measured factors, we will apply fully sequential multiple imputation to impute missing data [73], where the imputation models include all variables in the respective outcome models plus additional variables likely related to the risk of missingness or the values of the variables being imputed. Using multiple imputation will assure that statistical inferences are approximately unbiased if the mechanism of missingness follows a missing-at-random structure [74] after accounting for analysis variables and additional auxiliary variables in the imputation models.

### Sample Size Determination

Sample size estimation was based on the following assumptions: 1) 80% retention across the follow-up period; 2) a minimum clinically important difference for the PEG of 1.0 with a standard deviation of 2.5 [58, 60], which translates to a difference of 40% of 1 standard deviation between treatment groups; 3) serial correlations of *R* = 0 for repeated PEG assessments. With these assumptions, a sample size of 136 per group (total N = 272) provides 80% power with α* *= 0.05 between groups at the 26-week assessment (Aim 1). These calculations assume a zero correlation among the repeated measures. Because the PEG assessments at different time points are likely to be positively correlated, this power calculation is conservative, and the actual power to detect a difference of 40% of 1 standard deviation could exceed 80%.

## Discussion

Suboptimal clinical outcomes and persistent opioid use after lumbar surgery suggest that new, innovative pain management pathways might be needed. The MIND-PT trial examines an enriched pain management pathway that integrates PT with mindfulness techniques applied both before and after spine surgery. Mindfulness techniques in this study are derived from the MORE program, which has demonstrated efficacy for reducing chronic pain and opioid use [54] but to date has not been tested in combination with PT.

Physical therapists increasingly use techniques from interventions that integrate exercise with cognitive and behavioral strategies, including mindfulness techniques [75–77]. Mindfulness techniques help patients disentangle an experience (e.g., pain) from associated emotions and appraisals. Mindfulness can enhance emotion regulation and raise unconscious behavioral coping responses (e.g., opioid use) to conscious consideration. Helping patients develop nonjudgmental awareness reduces cognitive or behavioral reactivity to pain. These reactions facilitate central sensitization and amplify the pain experience, often leading to maladaptive coping strategies, such as reliance on pain medication. Mindfulness is a therefore a key way to cope with pain and avoid unhelpful coping strategies. Combining these techniques with exercise and the education typically provided after surgery has the potential to enhance the effectiveness of postsurgical rehabilitation and increase the likelihood of discontinuing opioids as a pain coping strategy.

There are several limitations in the design of the MIND-PT trial that should be acknowledged. The design does not attempt to balance the provider contact time between intervention arms. The long-term follow-up is 6 months after surgery. A longer-term follow-up would be beneficial to examine the persistence of any between-group differences. Providers of MIND-PT receive 1-day training to integrate mindfulness, and fidelity monitoring relies on provider self-report, which could limit the attribution of any observed between-group differences to specific elements of the study protocol [78]. Patient adherence is not assessed beyond session attendance.

In summary, the MIND-PT trial study will evaluate a novel pain management pathway for persons after lumbar spine surgery that integrates PT and mindfulness in an effort to address the cognitive and behavioral consequences of long-standing, presurgical chronic pain. In addition, this study will examine possible mechanisms through which the pathway might exert therapeutic effects, including pain catastrophizing and self-efficacy. We expect that the MIND-PT trial will provide important information to improve pain management after spine surgery.

## Funding

This work is supported by grant number 3UH3AT009763-04S1 from the National Center for Complementary and Integrative Health at the National Institutes of Health (NIH). The study is part of the Helping to End Addiction Long-term (HEAL) initiative as part of a group of studies within the NIH Back Pain Consortium (BACPAC) program. The content is solely the responsibility of the authors and does not necessarily represent the official views of the NIH.

*Conflicts of interest:* Dr. Garland reports significant financial interest (remuneration and licensed intellectual property) from BehaVR, a non–publicly traded company that has licensed the investigator's intellectual property being used in the study.

## Supplement sponsorship

This article appears as part of the supplement entitled “Back Pain Consortium (BACPAC) Research Program” sponsored supported by the NIH through the NIH HEAL Initiative under award number AR076730-01.

## Disclaimer

The view(s) expressed herein are those of the author(s) and do not reflect the official policy or position of Brooke Army Medical Center, the U.S. Army Medical Department, the U.S. Army Office of the Surgeon General, the Department of the Army, Defense Health Agency, Department of Defense, the Uniformed Services University, the U.S. Government, or the Henry M. Jackson Foundation for the Advancement of Military Medicine, Inc. The content is solely the responsibility of the authors and does not necessarily represent the official views of the NIH or its NIH HEAL initiative.

## Notes

Study registration: ClinicalTrials.gov ID NCT04770480.
